# Transmission and Toxigenic Potential of *Vibrio cholerae* in Hilsha Fish (*Tenualosa ilisha*) for Human Consumption in Bangladesh

**DOI:** 10.3389/fmicb.2018.00222

**Published:** 2018-02-20

**Authors:** Zenat Z. Hossain, Israt Farhana, Suhella M. Tulsiani, Anowara Begum, Peter K. M. Jensen

**Affiliations:** ^1^Department of Microbiology, University of Dhaka, Dhaka, Bangladesh; ^2^Institute of Public Health, University of Copenhagen, Copenhagen, Denmark; ^3^Copenhagen Centre for Disaster Research, University of Copenhagen, Copenhagen, Denmark

**Keywords:** fish, Hilsha, *Vibrio cholerae*, transmission, population, pathogenic potential, Bangladesh

## Abstract

Fish have been considered natural reservoirs of *Vibrio cholerae*, the deadly diarrheal pathogen. However, little is known about the role of fish in the transmission of *V. cholerae* from the Bay of Bengal to the households of rural and urban Bangladesh. This study analyzes the incidence and pathogenic potential of *V. cholerae* in Hilsha (*Tenualosa ilisha*), a commonly caught and consumed fish that exhibits a life cycle in both freshwater and marine environments in Bangladesh. During the period from October 2014 to October 2015, samples from the gills, recta, intestines, and scale swabs of a total of 48 fish were analyzed. The fish were collected both at local markets in the capital city Dhaka and directly from fishermen at the river. PCR analysis by targeting *V. cholerae* species-specific *ompW* gene revealed that 39 of 48 (81%) fish were positive in at least one of the sample types. Real-time PCR analysis demonstrated that the cholera-causing *ctxA* gene was detected in 20% (8 of 39) of *V. cholerae*-positive fish. A total of 158 *V. cholerae* isolates were obtained which were categorized into 35 genotypic groups. Altogether, 25 O1 and 133 non-O1/O139 strains were isolated, which were negative for the cholera toxin gene. Other pathogenic genes such as *stn/sto, hlyA, chxA, SXT, rtxC*, and *HA-P* were detected. The type three secretion system gene cluster (TTSS) was present in 18% (24 of 133) of non-O1/O139 isolates. The antibiotic susceptibility test revealed that the isolates conferred high resistance to sulfamethoxazole-trimethoprim and kanamycin. Both O1 and non-O1/O139 strains were able to accumulate fluid in rabbit ileal loops and caused distinctive cell death in HeLa cell. Multilocus sequence typing (MLST) showed clonal diversity among fish isolates with pandemic clones. Our data suggest a high prevalence of *V. cholerae* in Hilsha fish, which indicates that this fish could serve as a potential vehicle for *V. cholerae* transmission. Moreover, the indigenous *V. cholerae* strains isolated from Hilsha fish possess considerable virulence potential despite being quite diverse from current epidemic strains. This represents the first study of the population structure of *V. cholerae* associated with fish in Bangladesh.

## Introduction

Cholera, caused by *Vibrio cholerae*, remains a major burden in most Asian and African developing countries. Human cholera epidemics have been mostly caused by *V. cholerae* toxigenic serogroups O1 and O139 which express two principal virulence factors, cholera toxin (CT) and the colonization factor known as toxin-coregulated pilus (TCP) (Faruque et al., 1998; Harris, 2012). The other serogroups, collectively referred to as non-O1/O139 serogroups are mostly nonpathogenic, environmental isolates that express other O antigens (Dziejman et al., 2005). However, some non-O1/O139 *V. cholerae* are clearly pathogenic and responsible for acute cholera-like diarrhea (Ramamurthy et al., 1993; Sharma et al., 1998) and a variety of extra-intestinal infections (Morris Jr and Black, 1985). Despite the lack of cholera toxin, a few pathogenic non-O1 and non-O139 strains such as O141, O10 and O12 have caused outbreaks of gastroenteritis (Bagchi et al., 1993; Dalsgaard et al., 1995; Rudra et al., 1996). Recently, genomic analysis has demonstrated that non-O1/O139 strains contributed to the early cholera outbreak in Haiti as the sole pathogen for potentially a high proportion of cases (Hasan et al., 2012). However, studies have indicated that some of the potential virulence factors such as hemagglutinin protease, repeats-in-toxin, mannose-sensitive haemagglutinin, heat-stable enterotoxin, hemolysin and type III secretion system (T3SS) are essential for the diarrheagenic mechanism of non-O1/non-O139 (Nair et al., 1988; Thelin and Taylor, 1996; Rivera et al., 2001; Dziejman et al., 2005). Animal models have been extensively used to study the pathophysiology of diarrhea caused by CT of *V. cholerae* that adheres to human intestinal mucosa and induces an inflammatory response (De and Chatterje, 1953; Singh et al., 2001; Ritchie and Waldor, 2009). Furthermore, recent investigations also suggest that other non-CT virulence factors and inflammatory responses induced by *V. cholerae* independently of CT may contribute to the pathogenesis of cholera (Hodges and Gill, 2010; Chatterjee and Chaudhuri, 2013; Sawasvirojwong et al., 2013). Therefore, both O1/O139 and non-O1/O139 serogroups of *V. cholerae* pose considerable threat to public health.

Bangladesh is an area of cholera endemicity where this disease occurs in seasonal regularity with more than 100,000 cases annually (Lipp et al., 2002; Ali et al., 2015). An annual single peak of cholera cases (March-May) is observed in rural coastal villages, whereas cholera outbreaks maintain a unique bimodal seasonality in the capital city of Dhaka, where the larger peak with the highest number of cases occurs just after the monsoon (September–November), with a smaller peak in the spring (March–May) (Kaper et al., 1995; Faruque et al., 2005; Alam et al., 2011). Major cholera outbreaks primarily originated in the coastal regions of southern Bangladesh, including the initial appearance of O139 Bengal in the coastal areas in 1992, which then spread inland through secondary means (Jutla et al., 2010). Isolation of pandemic strains from the aquatic environment of endemic regions, even during seasonal outbreaks, is rare because toxigenic strains may persist in a non-culturable state (Brayton et al., 1987; Alam et al., 2006). *Vibrio cholerae* may enter into a viable but non-culturable (VBNC) state to persist in the stressed conditions of aquatic environments, in which they may not form colonies on traditional bacteriological culture media (Alam et al., 2007). It is likely that the environment is the source of epidemic strains; however, the mechanism that enables spreading of *V. cholerae* across water bodies from the Bay of Bengal is still not clearly understood (Halpern et al., 2008). It has been suggested that *V. cholerae* proliferates while attached to planktonic bodies, particularly copepods, in aquatic systems (Huq et al., 1983; Colwell, 1996). Migratory water birds and fish have also been linked to *V. cholerae* dissemination between water bodies of western Asia, Europe and Africa (Halpern et al., 2008; Senderovich et al., 2010; Halpern and Izhaki, 2017). In Bangladesh, recurrent cholera infections have been linked to increased environmental concentration of plankton in river delta, although, a recent study has shown the absence of a direct connection between the riverine system and drinking water sources (Grant et al., 2015). Precisely, the transmission of *V. cholerae* between the Bay of Bengal and a major city like Dhaka still remains unknown.

**Figure 1:**
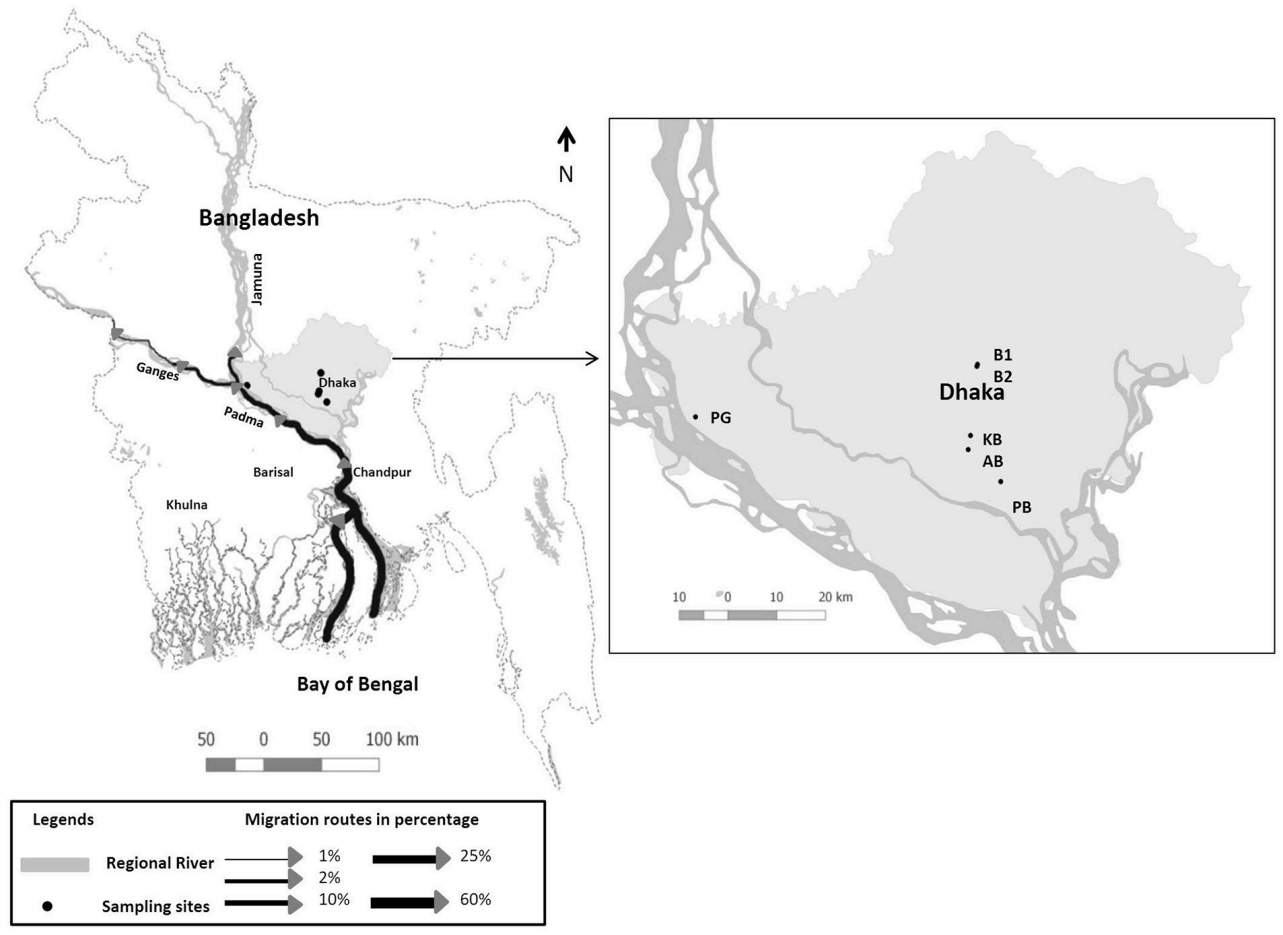
Map showing Hilsha migration routes in Bangladesh adopted from Ahsan et al. (2014) and sample collection sites are indicated: Boubazar 1(B1), Boubazar 2 (B2), Kawran Bazar (KB), Ananadabazar (AB), Pagla bazaar (PB), and Paturiaghat (PG).

It was postulated that Hilsha fish (*Tenualosa ilisha*), which migrates between both coastal and up-stream freshwater environments for breeding, might play a role in maintaining cholera endemicity in India (Pandit and Hora, 1951). The authors, however, failed to undertake any direct field or laboratory investigations to validate their hypothesis. Hilsha (*Tenualosa ilisha)* is anadromous in nature, migrating from the Bay of Bengal to inland freshwater through rivers on the Indian sub-continent for spawning, which occurs from July to October and again from January to March (Ahsan et al., 2014) (see Figure 1). The upstream migration of Hilsha is associated with the state of sexual maturity, as well as the volume of freshwater discharge from the estuary during monsoons (Bhaumik, 2017). The catch percentage of Hilsha is very high during these migratory periods. Approximately 300,000 tons of Hilsha were caught in inland and marine waters in Bangladesh from 2010 to 2011 (Ahsan et al., 2014). The availability of the fish in local markets also increased during these periods, which results in lower prices. During the remainder of the year and national festivals such as the Bengali New Year, the fish is usually too expensive for poor communities.

In this study, Hilsha fish is analyzed for the first time to be a potential carrier of *V. cholerae*, and for its role as a risk factor in the transmission of *V. cholerae* to humans. The rationale of the current study is to analyze the incidence and seasonal prevalence of *V. cholerae* in Hilsha from both the Padma River, where they have migrated from the Bay of Bengal, and in local markets in Dhaka. The study extensively characterizes the population structure of *V. cholerae* strains isolated from fish, and assesses the pathogenic potential of these strains. In addition, the clonal relationship between environmental and endemic strains was analyzed to discern the understanding of evolutionary history. The study was conducted as a part of a research project funded by the Danish Government (DANIDA) called “Combating Cholera Caused by Climate Change” (C5). The project focuses on the cholera influencing factors by identifying relative risks based on environmental and hygienic issues in Bangladesh (http://cope.ku.dk/research/cholera/).

## Materials and methods

### Sample collection and processing

Four Hilsha fish were collected each month for a period of 1 year, from October 2014 to September 2015; two “market fish” from local markets in and around Dhaka and two “fresh fish” which were freshly caught near the bank of the Padma River (for a total of 48 fish) Figure 1, Supplementary Table 1. All the fish (mean body weight 765 g) were healthy, with bright, shiny appearance and natural odor.

The fresh fish were bought directly from fishermen at the major landing point (Paturia, approximately 80 km away from Dhaka) early in the morning. The fish were caught on the previous night in the lower Padma River between Shariatpur and Chandpur. Each fish was collected in individual sterile collection bags and transported to the University of Dhaka laboratory within 4 h of collection in a cool box maintaining cold condition. Four samples were aseptically taken from each fish:—two slits of fish gills, gut, rectum and an outer swab of scales in phosphate-buffered saline (PBS). For the fish collected from the local market (fish stored on ice), ice samples where the fish were kept frozen were also collected in sterile zip-lock sample collection bags. A total of 8 and 10 samples each of fresh-caught fish and local market fish were analyzed each month for 1 year, for a final total of 96 and 120 samples. Approximately 6 gm of gill, gut and rectum samples were enriched in 60 mL of Alkaline Peptone Water (APW) (1 L distilled H_2_O, 10 gL^−1^ peptone, 10gL^−1^ sodium chloride; pH 8.5). One mL of PBS outer swab and storage ice water were transferred to 9 mL of APW for enrichment. All of the samples were incubated at 37°C for 24 h.

### Total DNA extraction and detection of *V. cholerae* by polymerase chain reaction

Total DNA was extracted from all of the samples using the boiled template method (De Medici et al., 2003). The presence of *Vibrio cholerae* in total DNA of fish samples was confirmed by PCR using the previously published primers (5′-CACCAAGAAGGTGACTTTATTGTG-3′ and 5′-GGTTTGTCGAATTAGCTTCACC-3′) for the outer membrane protein (*ompW*) gene of *V. cholerae* (Nandi et al., 2000).

The PCR was conducted in a thermal cycler (MJ Research PTC-200, USA) using 0.2 mL PCR tube with a reaction volume of 12 μL containing 1 μL of 10X PCR buffer including 20 mM MgCl_2_, 0.2 μL of 10 mM deoxynucleoside triphosphates (dNTP) mix (Thermo Scientific, USA), 0.05 μL of 5 U Dream Taq DNA Polymerase (Thermo Scientific, USA) per μL, 0.625 μL of 25 μM each primer (Tag Copenhagen A/S, Denmark), 7.5 μL of nuclease-free water and 2 μL of DNA template. The PCR tubes containing reaction mixtures were heated at 95°C for 3 min for complete denaturation of DNA templates. The PCR amplification was carried out for 35 cycles in the following order: initial denaturation at 95°C for 45 s, annealing at 55°C for 45 s, hybridization at 72°C for 45 s, with a final extension at 72°C for 7 min. PCR products (304 bp band size) were then resolved by 1.5% (wt/vol) agarose gel electrophoresis and visualized with a UV transilluminator (Gel Doc, Bio-Rad, USA) after ethidium bromide staining.

The chance of contamination between local market fish and fresh-caught fish was scored by the presence or absence of specific *ompW* targets and was statistically analyzed by Fisher's exact test for a 2 × 2 contingency table in statistical software R version 3.3.1. Significance was defined as having a *p* value of less than 0.05.

### Bacterial strains

A total of 158 *V. cholerae* strains were isolated by using conventional cultural media TCBS (Thiosulfate citrate bile-salts sucrose agar). Species identification of all the strains was further confirmed by standard biochemical assays and *V. cholerae* species-specific *ompW* gene target PCR (Nandi et al., 2000; Choopun et al., 2002; Huq et al., 2012).

### Molecular characterization of *V. cholerae* isolates

Genomic DNA from the isolates was extracted by the boiled template method described earlier. Serological assays and PCR targeting the *rfb* sequences specific for O1 and O139 serogroups were used for further subtyping of all *V. cholerae* isolates. PCR was performed to detect the virulence and regulatory genes of *V. cholerae* O1/O139 and non O1/O139 (Supplementary Table 2). Total DNA samples that were detected as *V. cholerae*-positive were further analyzed for the presence of cholera toxin gene (*ctxA*) and the *rfb* sequences of O1 and O139 serogroups. PCR reactions were performed by using the protocol described previously in the section Total DNA Extraction and Detection of *V. cholerae* by Polymerase Chain Reaction. The primers, probes used in this study are listed in Supplementary Table 2. Real time PCR to detect *ctxA* gene was performed by following the previously published protocol (Blackstone et al., 2007). Positive and negative controls used in PCR experiments are listed in Supplementary Table 3.

### *rpoB* sequencing

Species identities of representative 36 *V. cholerae* strains were confirmed by nucleotide sequencing of 871 bp fragment of the *rpoB* gene. PCR based amplification and sequence analysis of *rpoB* gene were conducted as described previously (Tarr et al., 2007). For sequencing, BigDye Terminator v3.1 sequencing kit (Applied Biosystems, USA) was used following manufacturer's instructions. Sequence determination was conducted onABI3730XL (Applied Biosystems, USA) system.

### Antibiotic susceptibility assay

Antibiotic susceptibility of the *V. cholerae* strains was conducted by agar disk diffusion method using commercial disks (Oxoid, UK). The strains were tested for Tetracycline (30 μg), Sulfamethoxazole-trimethoprim (25 μg), Chloramphenicol (30 μg), Kanamycin (30 μg), Neomycin (30 μg) according to the standard guidelines of Clinical and Laboratory Standards Institute (CLSI) (Patel et al., 2014). The zone standards for *Enterobacteriaceae* were used when there were no established breakpoint interpretive criteria for *V. cholerae*. *E. coli* ATCC 25922 was used as quality control strain. The experiment was done in duplicate.

### Toxicity assay

Nine *V. cholerae* strains were studied including 6 *V. cholerae* O1 and 3 non O1/O139 serogroups (Table 3) for analyzing pathogenic potential on established animal model and human cancer cell line. Multilocus sequence typing (MLST) method was used to determine the nucleotide changes in housekeeping genes of these 9 isolates compared to existing database (see section MLST below).

#### Tissue culture assay

Culture supernatants of *V. cholerae* strains were tested for cytotoxicity in HeLa cell-line (human cervical carcinoma cell-line). Following previous protocol, the cell-free culture supernatants were prepared by centrifugation and filtration through a 0.22-μm-pore size filter unit (Millex-GS; Millipore Corp., Bedford, Mass; Sharma et al., 1998).

HeLa cells were grown as monolayers in Dulbecco's Modified Eagles' medium (DMEM) (Thermo Fisher Scientific, USA) containing 1% penicillin-streptomycin (1:1) and 0.2% gentamycin and 10% fetal bovine serum (FBS). Cells (4.4 × 10^4^/400 μl) were seeded onto 24-well plates and incubated overnight at 37°C in a humidified 5% CO_2_ atmosphere. Thereafter, 100 μl of the culture supernatant sample was added each well. Cytotoxicity was examined under an inverted light microscope (Olympus, Japan) after 24 h of incubation. The uninoculated Trypticase soy broth and *V. cholerae* O1 El Tor N16961were used as negative and positive control. Duplicate wells were used for each sample.

#### Rabbit ileal loop assay

Cultures of *Vibrio cholerae* were tested for ileal loop fluid accumulation in adult New Zealand albino rabbits as described (De and Chatterje, 1953). The experiments were performed at International Centre for Diarrheal Disease (icddr, b) Bangladesh in complete accordance with icddr, b ethical guidelines. The protocol was reviewed and approved by icddr, b “Animal Experimentation Ethics Committee (AEEC).” Each test was done in duplicate (in two rabbits). Toxigenic *V. cholerae* N16961 and PBS were used as positive and negative control respectively. *V. cholerae* strains with little or no fluid accumulation in the initial passage were recovered from the ileal loops on nutrient agar plates and subjected to second passage in the same way by using the protocol of Sanyal et al. (1984). This process was repeated until third passage to obtain unambiguous positive response.

### Multilocus sequence typing

Seven house-keeping genes (*adk, gyrB, mdh, metE, purM, pntA*, and *pyrC*) were recovered by PCR from all nine strains and the products were sequenced. The primer sequences were extracted from previously published work (Octavia et al., 2013). Homologous sequences from these seven loci were sourced from database entries of whole and partial sequences with the following genome strains and accession nos.- N16961 (Accession No.AE003852); BX330286 (Accession No. ACIA00000000); MZO-3 (Accession No. AAUU00000000); M2552 (KC894993, KC895055, KC895117, KC895179, KC89524, KC895303, KC895365); M2554 (KC894995, KC895057, KC895119, KC895181, KC895243, KC895305, KC895367); M1619 (KC894986, KC895048, KC895110, KC895172,KC895234, KC895296, KC895358). Sequences for the fish isolates are deposited in GenBank under accession nos. KY619689–KY619697 (*adk*), KY619698–KY619706 (*gyrB*), KY619707–KY619715 (*mdh*), KY619716–KY619724 (*metE*), KY619725–KY619733 (*pntA*), KY629640–KY629648 (*purM*), KY629649–KY629657 (*pyrC*).

A multiple alignment of sequences generated by the study and those extracted from the Genbank database was constructed using MAUVE software package (http://asap.ahabs.wisc.edu/software/). The aligned file was used as input for Bayesian inference of genealogy and recombination events using CLONALFRAME v. 1.2 software following the published method along with model parameters (Didelot and Falush, 2007; Islam et al., 2013). The number of populations was determined by the Markov Chain Monte Carlo (MCMC) simulation of 10,000 iterations which gave the posterior probability of *K* following a burn-in of 10,000 iterations and parameter values were recorded for 10 iterations in the posterior sample. Analysis was repeated three times with same data and parameters, but with distinct starting points and 50% consensus trees were produced by Clonal Frame with a threshold of 0.5. The relative effect of homologous recombination on the genetic diversification of populations was measured by calculating the ratio of recombination and mutation events (r/m) (Guttman and Dykhuizen, 1994).

### Ethics statement

This study was undertaken in accordance with the ethical recommendation of Faculty of Biological Sciences, University of Dhaka, Bangladesh. All the fish for this study were obtained directly from fishermen and fish mongers in local markets selling for consumption. The fish were not alive during the time of collection.

## Results

### Prevalence of *V. cholerae* in fish samples

**Figure 2:**
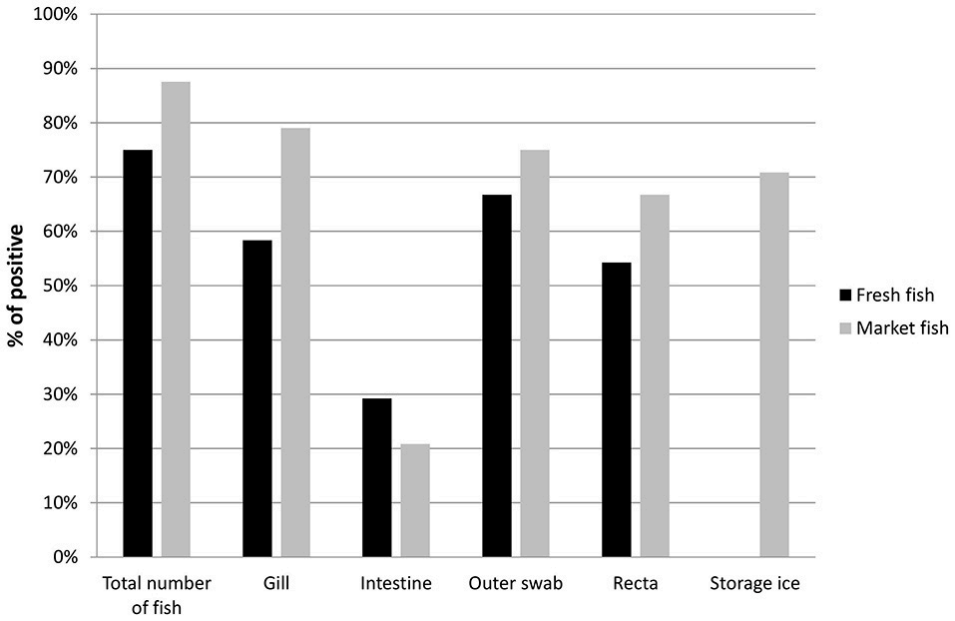
Prevalence of *V. cholerae* in total number of fish (Fresh fish, *n* = 24 and Market fish, *n* = 24) and different fish parts (no ice was collected for any of the fresh fish). Positivity of *V. cholerae* was determined by *ompW* gene target PCR.

Of the total of 48 individual fish (216 total DNA samples) collected, 39 (81%) fish were positive for the specific *ompW* gene when assayed for *V. cholerae*. Among the market fish, detection was highest in the gills (19 of 24 fish, 79%), followed by outer scale swabs, recta and intestines. In fresh fish, detection was highest in outer scale swabs (16 of 24 fish, 66.7%), followed by the gills, recta and intestines (Figure 2). Seventeen storage ice samples were positive for *V. cholerae* out of 24 (70.8%) market fish by PCR. No local market fish was found to be positive only for ice water. The presence of *V. cholerae* was higher in fish purchased from local markets (21 of 24, 87.5%) by PCR than fish from the river banks (18 of 24, 75%), where the fish were considered positive if any part of the fish was positive. A total of 55% (53 of 96) of fresh fish sample types and 60% (72 of 120) of local fish sample types including ice samples were found to be positive for *V. cholerae*.

The prevalence of *V. cholerae* contamination was statistically compared between the fish types. Statistical analysis using Fisher's exact test yielded that the chance of *V. cholerae* contamination in market fish stored on ice is higher than in fresh fish and showed a significant difference (odds ratio [OR]: 0.41; 95% confidence interval [CI]: 0.2, 0.9; *P* = 0.03). No statistically significant difference was found (*P* = 0.6) when the analysis was done on total individual samples (96 from fresh fish and 120 from local market fish).

### Molecular genotyping of total DNA and monthly incidence

Total DNA samples were extracted from Hilsha fish and ice samples for *V. cholerae* detection and isolation. Among them, 125 (58%) samples tested positive based on the *ompW* PCR. The *ompW* positive total DNA samples were further characterized by targeting genes of toxigenic *V. cholerae*, which yielded 25 positive amplicons (16 fish) for the *V. cholerae* O1 serogroup and 9 (6 fish) for the O139 serogroup. Seven of the *V. cholerae* O1-positive and three of the O139-positive samples were also positive for *ctxA* in real-time PCR which comprised 8% of all *V. cholerae*-positive samples and 20% of all *V. cholerae*-positive fish. Detection of *V. cholerae* O1, O139 and *ctxA* genes was compared between the two types of fish, and 23.8% (5 out of 21) of local market fish were found to be positive for the *ctxA* gene, an amount that was higher than for fresh fish samples (16.67%, 3 out of 18). The results are shown in Table 1. The detection of *rfbO1* and *ctxA* gene was confirmed as positive in 11.8% (2 out of 17) and 5.9% (1 out of 17) *ompW* positive storage ice samples respectively and none were positive for *rfbO139* gene. One market fish was found *ctxA* gene positive in ice only, whilst the other parts were negative.

**Table 1:** Occurrence of toxigenic *V. cholerae* genes in fresh and market fish.

**Fish types**	**No. of *V. cholerae* positive fish/total (%)**	**No. of *V. cholerae* positive samples /total (%)**	**No. of *V. cholerae* O1 positive fish/total (%)**	**No. of *V. cholerae* O1 positive samples/total (%)**	**No. of *V. cholerae* O139 positive fish/total (%)**	**No. of *V. cholerae* O139 positive samples/total**	**No. of *ctxA* positive fish/total (%)**	**No. of *ctxA* positive samples/total (%)**
Fresh fish	18/24 (75)	53/96 (55.2)	6/18 (33.3)	8/53 (15)	2/18 (11.1)	3/53 (5.7)	3/18 (16.67)	4/53 (7.5)
Market fish	21/24 (87.5)	72/120 (60)	10/21 (47.6)	17/72 (23)	4/21 (19)	6/72 (8.3)	5/21 (23.8)	6/72 (8.3)

**Figure 3:**
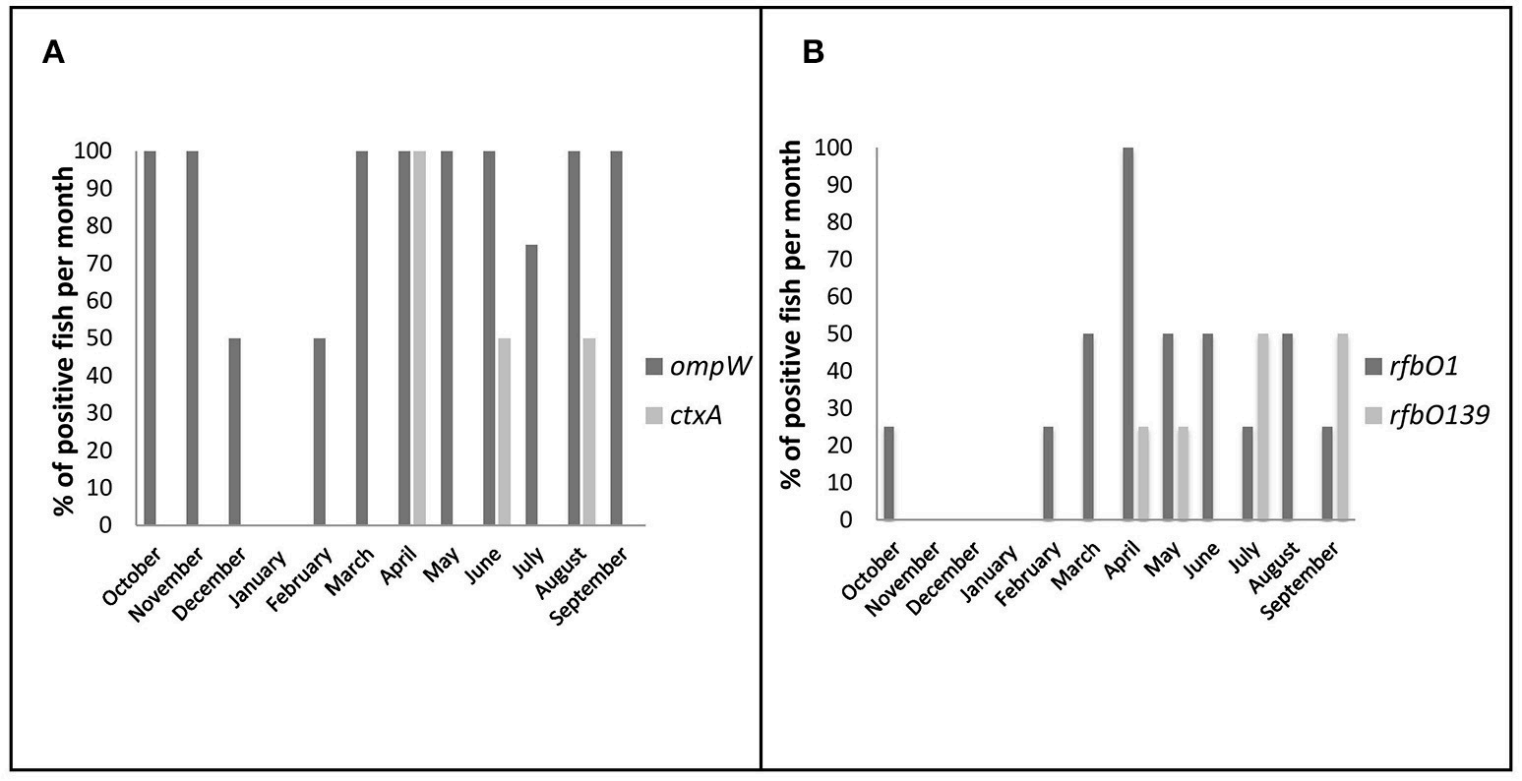
Seasonal variation of total and toxigenic *V. cholerae* prevalence in direct DNA samples (%, bar chart). The fish was considered as positive when any part of the fish was found positive in PCR (including ice samples of market fish). **(A)** Positive fish per month for *ompW* and *ctxA* genes **(B)** Positive fish per month for toxigenic serogroups O1 and O139.

The monthly incidence of *V. cholerae* in fish revealed two annual peaks; one from March to June before the monsoon, followed by a second peak in August-November at late monsoon (Figure 3). Detection of DNA from toxigenic *V. cholerae* O1 and cholera toxin peaked in April when 100% (4 of 4) of the fish were positive for both the *rfbO1* and *ctxA* genes. Detection of *V. cholerae* O139 was highest in July and September when 50% (2 of 4) of all the fish were positive for the *rfbO139* gene.

### Genotyping of *V. cholerae* strains

A total of 158 strains isolated from fish were confirmed as *V. cholerae* species by cultural and biochemical tests and *ompW* gene specific PCR. A total of 35 groups of 158 strains were detected using PCR based genotypic characterization (Supplementary Table 2). Twenty-five *V. cholerae* strains were positive for the *rfbO1* gene and 133 strains were negative (non O1/O139), which are included in 13 and 23 groups respectively (Table 2). None were positive for the *rfbO139* gene and none of the O1 strains contained cholera toxin genes A and B (*ctxA* and *ctxB*), the toxin-coregulated pilus (*tcp*), colonization factor (*ace*), and core-encoded pilus (*cep*) genes. The highest numbers of isolates (29) in our study were categorized in Group-XXIX with a genotypic trait of *rfbO1*^−^*chxA*+*mshA*+*ompU*+. The heat stable enterotoxin of *V. cholerae* (*stn/sto*) was present in three out of 25 O1 strains and four out of 133 non-O1 strains. We studied the SXT mobile genetic element that encodes antibiotic resistance in all the strains, and 37 strains (4 O1 and 33 non O1/O139) were positive. Twenty-four (18%) non-O1 strains were positive by PCR for all three genes (*vcsN2, vcsC2, vopF*) tested for type three secretion system. Sixty-eight percent O1 and 80% non-O1 experimental strains possessed mannose sensitive hemagglutinin pilus (*mshA*). Previous studies show that newly discovered Cholix toxin (*chxA*) is mostly found in non O1 *V. cholerae* (Awasthi et al., 2013). However, approximately 80% of the O1, and 71% of the non O1 strains of this study were positive for the *chxA* gene. The gene of the putative outer membrane protein (*ompU*) was found in 14 (56%) O1 and 62 (47%) non-O1 strains. All the strains were positive for hemolysin hlyA, hemagglutinin protease (HA- protease), toxR, rtxC, and the type six secretion system (vasA, vasH, vasK) PCR. Isolation of *V. cholerae* dropped in January and remained the same for the next 2 months.

**Table 2:** Grouping of *V. cholerae* isolates based on genotypic characterization.

**Group**	**No. of Isolates**	***hlyA, HA protease, toxR, rtxC*, T6SS**	***rfbO139, ctxA, ctxB, cep, tcp, zot*, *ace***	***rfbO1***	**T3SS *(vcsN2, vcsC2, vcsC2)***	***mshA***	***chxA***	***ompU***	***stn/sto***	***SXT***
I	1	+	−	+	−	+	−	−	+	−
II	2	+	−	+	−	+	+	+	+	−
III	1	+	−	+	−	+	−	−	−	+
IV	2	+	−	+	−	+	+	+	−	+
V	1	+	−	+	−	−	+	+	−	+
VI	5	+	−	+	−	+	+	+	−	−
VII	3	+	−	+	−	−	+	+	−	−
VIII	2	+	−	+	−	−	+	−	−	−
IX	4	+	−	+	−	+	+	−	−	−
X	2	+	−	+	−	−	−	−	−	−
XI	1	+	−	−	−	+	−	+	+	−
XII	2	+	−	−	−	+	+	+	+	−
XIII	1	+	−	−	−	−	−	−	+	+
XIV	10	+	−	−	−	+	+	+	−	+
XV	14	+	−	−	−	+	+	−	−	+
XVI	3	+	−	−	−	−	+	−	−	+
XVII	3	+	−	−	−	−	−	−	−	+
XVIII	1	+	−	−	−	+	−	−	−	+
XIX	1	+	−	−	+	+	+	−	−	−
XX	1	+	−	+	+	+	+	+	−	−
XXI	1	+	−	+	+	+	−	−	−	−
XXII	1	+	−	−	+	+	+	+	−	+
XXIII	3	+	−	−	+	+	+	+	−	−
XXIV	6	+	−	−	+	+	+	+	−	−
XXV	1	+	−	−	+	+	+	−	−	−
XXVI	1	+	−	−	+	−	−	+	−	−
XXVII	5	+	−	−	+	+	−	−	−	−
XXVIII	4	+	−	−	+	−	+	−	−	−
XXIX	29	+	−	−	−	+	+	+	−	−
XXX	14	+	−	−	−	+	+	−	−	−
XXXI	7	+	−	−	−	−	−	−	−	−
XXXII	3	+	−	−	−	+	−	+	−	−
XXXIII	6	+	−	−	−	−	+	+	−	−
XXXIV	1	+	−	−	−	−	+	−	−	−
XXXV	16	+	−	−	−	+	−	−	−	−
	Total = 158	158		25	24	123	115	76	7	37

A total of 35 representative *Vibrio cholerae* strains of 35 genotypic groups were sequenced to identify those that showed different molecular characteristics. The partial nucleotide sequences of the *rpoB* gene have been evaluated for species identification of *V. cholerae*. The alignment of the study sequences to databases using BlastN (http://blast.ncbi.nlm.nih.gov/Blast.cgi) confirmed 99–100% sequence similarity with *V. cholerae* species. Isolate information and accession numbers are listed in Supplementary Table 4.

### Antibiotic sensitivity test

**Figure 4:**
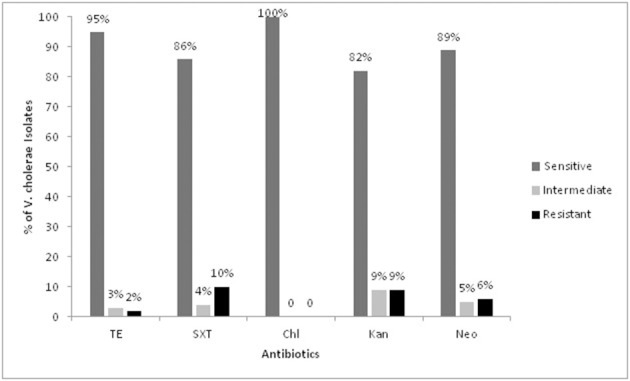
Antibiotic susceptibility pattern of the *Vibrio cholerae* isolates. TE, Tetracycline; SXT, Sulfomethoxazole-trimethoprim; Chl, Chloramphenicol; Kan, Kanamycin; Neo, Neomycin.

All the *V. cholerae* strains (*n* = 158) were tested for their antibiotic susceptibility against five commonly prescribed antibiotics. The antibiotic response of the strains revealed that all were uniformly susceptible to Chloramphenicol (100%). Two (1%), 17 (10%), 15 (9%) and 10 (6%) isolates were found to be resistant to tetracycline, Sulfamethoxazole-trimethoprim, kanamycin, and neomycin respectively. Thirty-five isolates showed antimicrobial drug resistance to at least one of the test antibiotics. All these strains carried the gene for the SXT mobile genetic element. The results are shown in Figure 4.

### Pathogenicity assays

Cell-free culture supernatants of nine *Vibrio cholerae* isolates caused morphological changing from cell rounding to cell clumping and cell death of the HeLa cell line. Six of the nine isolates were cytotoxic, three of these (F-44, F-52a, F-91b) induced massive cell death (<1% survival of HeLa cells), indicating the presence of extracellular cytotoxic proteins. The severity of cytotoxicity was relatively less apparent in the supernatants of F-45 and 49d. Three isolates, F-36a, F-47, and F-53 exhibited negligible cytotoxicity with survival of more than 90% of HeLa cells. Morphological changes induced by cell-free culture supernatants of *V. cholerae* were detected by microscopic examinations. Compared to the fish isolates, the positive control *V. cholerae* O1 N16961 in this assay showed a lower cytotoxic effect.

All nine isolates were tested for their ability to cause fluid accumulation in the rabbit ileal loop model. All strains except F-36a had an FA index above the borderline of 0.5, the accepted cut-off for FA in diarrheagenic bacterial strains (Wallis et al., 1986; Islam et al., 2013). Six isolates caused fluid accumulation (FA index, 0.5–1.8) in the initial passage (Table 3). Isolate F-45 showed a positive response after three passages. The control strains N16961 showed a mean fluid accumulation of 1.6 mL/cm.

**Table 3:** Results of pathogenicity assays.

**Isolate ID (genotypic group)**	**Fish part from where isolated**	***rfb O1***	**Cytotoxic effect (survival of HeLa cells)**	**Rabbit ileal loop assay**
				Mean FA index (V/L)^*^
F-32b (I)	Outer surface	+	Cytotoxic (10–30%)	1.78 (1)
F-36a (VII)	Gill	+	Non cytotoxic (>90%)	0.46 (3)
F-44 (VIII)	Gut	+	Cytotoxic (<1%)	1.29 (1)
F-45 (IX)	Gill	+	Cytotoxic (<40%)	0.67 (3)
F-47 (XXIII)	Storage ice	–	Non cytotoxic (>95%)	0.87 (2)
F-49d (XIX)	Storage ice	–	Cytotoxic (20–50%)	0.53 (1)
F-52a (XXVI)	Rectum	–	Cytotoxic (<1%)	1.44 (1)
F-53 (IX)	Outer surface	+	Non cytotoxic (>95%)	1.73 (1)
F-91b (VIII)	Outer surface	+	Cytotoxic (<1%)	1.25 (1)

* *V, Volume of fluid in mL; L, Length of the loop in cm. Number of passages in bracket*.

*Positive cut-off value = 0.5 mL/cm*.

### Multilocus sequence typing analysis

**Figure 5:**
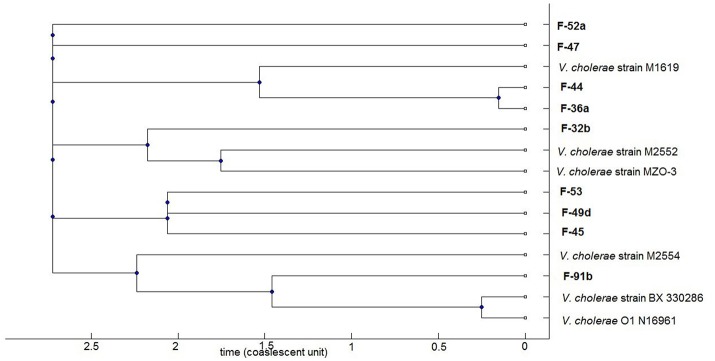
Phylogenomic tree based on concatenated multilocus sequences of the *V. cholerae strains*. The current study *V. cholerae* strains are highlighted as bold which comprises both O1 and non-O1 strains. The remaining strains representing clinical and environmental toxigenic O1 and non-O1 reference strains: strain M1619, environmental toxigenic non-O1; strain M2552, M2554, and MZO-3, clinical non-O1; strain BX330286, environmental toxigenic O1; strain N16961, clinical O1.

A consensus tree of genealogy was constructed by ClonalFrame software which demonstrates clonality of the population data (Figure 5). The fish isolates clustered into four major clades; of which three clades comprised of draft sequences from database as neighbor. One of these groups consists of isolate F-91b, *V. cholerae* strain N16961 and BX 330286, both of the database strains are toxigenic O1 El Tor but interestingly, BX 330286 was isolated from water samples in Australia. Another group comprises of *V. cholerae* strain M2552, MZO-3 and F-32b.The first two strains are clinical non-O1 isolates but the fish strain contains *rfbO1* gene but lacks *ctxA* gene. The third clade comprises F-36a, F-44, and *V. cholerae* strain M1619; the former two fish strains have almost similar virulence gene profile since F-44 only lacks *ompU* gene. Strain M1619, a non-O1/O139 *V. cholerae*, was recovered from environment in Australia and identified as carrying the VPI (*Vibrio* Pathogenicity Island) and CTX phage region. The cluster formation in sets of sequence types predicts the occurrence of recombination or point mutation in different alleles. Our analysis implies recombination events occur more often relative to mutation in study population. In the case of the seven genes analyzed here, the ratio of probabilities of nucleotide substitution through recombination and mutation (r/m) is 0.4 that means recombination induces evolution events 0.4 times higher than point mutations.

## Discussion

To the best of our knowledge, this is the only study on *V. cholerae* occurrence in Hilsha, a fish that migrates from cholera-endemic coastal waters upstream to freshwater rivers running through Bangladesh. Here, we show the population dynamics of *V. cholerae* strains isolated from freshly caught and local market Hilsha fish of Bangladesh and map their virulence profile and toxigenic potential. This study is the first to profile virulence related genes of *V. cholerae* in Hilsha fish.

Our results suggest that Hilsha fish may serve as a possible transmission vehicle of *V. cholerae* from the Bay of Bengal to inland Bangladesh due to their unique survival practices, such as migration for breeding and planktonic food. Previously, Bhuyan et al. (2016) reported the role of flooding in the seasonal dissemination of *V. cholerae* from coastal to inland water bodies in India, which corresponds with our investigation as the main period of Hilsha migration is during flooding caused by monsoon rains (Islam et al., 2016). Unlike the study conducted by Bhuyan et al. (2016), river water contamination was not analyzed in this study. Although there was a limited number of fish analyzed in our preliminary study, the data indicate that the occurrence of *V. cholerae* in Hilsha fish maintains similarity with the seasonal regularity of cholera epidemics in Bangladesh. The detection of *ctxA*-positive samples was highest in April. The presence of non-toxigenic O1/O139 and non-O1/O139 in fish may also play a critical role in cholera evolution and transmission, as they share the same environmental habitats as O1/O139 serogroups (Azarian et al., 2016). A higher occurrence of toxigenic *V. cholerae* genes is seen in the local market fish. A possible explanation for this observation is that unhygienic conditions for fish storage could make the fish more prone to cross-contamination with fecal matter.

*Vibrio cholerae* has been isolated from other fish species including fresh water *Tilapia* species in Israel, Burkina Faso, and Tanzania (Senderovich et al., 2010; Traor et al., 2014; Hounmanou et al., 2016), from Ayu fish in the rivers of Japan (Kiiyukia et al., 1992), from the species *Rastrineobola argentea* and *Oreochromis niloticus* in Lake Victoria, Kenya (Onyuka et al., 2011), and from ornamental fish in Czech Republic (Rehulka et al., 2015). Reports also demonstrate isolation of *V. cholerae* from marine fish species (Scheelbeek et al., 2009; Senderovich et al., 2010). Furthermore, Mrityunjoy et al. (2013) showed elevated bacterial load in frozen fish collected from Dhaka city in Bangladesh. So far, no studies have been undertaken to investigate the bacterial genomic characteristics isolated from fish of the Ganges Delta region and Bangladesh, where cholera is endemic.

Fish has been indicated as the source of cholera outbreaks in different continents. Cholera has been associated with consumption of raw fish and seafood (McIntyre et al., 1979; Maggi et al., 1997; Forssman et al., 2007). A cholera patient was identified in Berlin, who had become infected while handling and preparing imported fish from Nigeria (Schürmann et al., 2002). Although, fish accounts for approximately 66% of total animal food intake in Bangladesh (Belton et al., 2011), there is no study to our knowledge that examined fish as a transmission risk factor for cholera outbreaks. Hilsha (*Tenualosa ilisha*) is the most important fish species in Bangladesh, which alone contributes to more than 10% of the total fish catch (Ahsan et al., 2014).

A low-income area near Dhaka was selected in which to directly contact households for information on where they purchase their fish. Fishmongers were also asked about the source of their fish. In Bangladesh, fish are normally bought whole without cleaning and taken home to be gutted and cleaned by members of the household themselves. The gutting and slicing of fish normally occurs on the kitchen floor with a water source nearby. Lack of proper hand washing and reusing water for cleaning cutting materials is also observed in overcrowded urban communities with mixed incomes. Factors such as shared cooking areas and inadequate drainage systems lead to susceptibility to cholera infection in these neighborhoods (Wahed et al., 2013). This combination of attributes has been previously reported in a study in Monrovia, Liberia, which suggested a cholera transmission pattern based on the cleaning of fish, rather than its consumption (Scheelbeek et al., 2009).

*Vibrio cholerae* was isolated from 35 of 40 fish (115 of 125 positive fish specimen types) to test for *V. cholerae* specific PCR. Multiple isolates with different cultural and genotypic properties have been isolated from 6 fish (14 fish specimens) and isolation was not successful for 5 *V. cholerae* positive fish (10 fish specimens). The strains successfully isolated in this study were of nontoxigenic O1 and non-O1/O139 serogroups. None of the O1 isolates carried the genes for the major toxin genes *ctxA* and *tcp*, to which the clinical state of cholera is primarily attributed. Previous studies have shown that the O1 serogroup of *V. cholerae* frequently isolated from the aquatic environment commonly lack cholera toxin genes (Igbinosa and Okoh, 2008). In our study, the presence of the *ctxA* gene in direct DNA samples has been observed, but we have not succeeded in isolating these pathogenic strains. Difficulties in culturing cholera bacteria from environmental samples have been reported in previous studies. However, it has been shown that on average, culturing yielded positive results for only 1% of the environmental samples analyzed during epidemic periods, and rarely during interepidemic periods as cells enter into a viable but non-culturable (VBNC) state (Huq et al., 1990; Alam et al., 2006; Du Preez et al., 2010; Bhuyan et al., 2016). Fluctuations of environmental factors and the abundance of nontoxigenic isolates in the aquatic system may have an impact on the isolation of pathogenic strains (Mishra et al., 2012). It is noteworthy that small sample size and limited fish collection points could also be limiting factors in this study.

Toxigenic non-O1/O139 serogroups have caused severe cholera-like outbreaks in India and other countries, including Haiti (Rudra et al., 1996; Dalsgaard et al., 1999; Onifade et al., 2011; Hasan et al., 2012). Two toxigenic *V. cholerae* O1 strains, positive for cholera toxin, have been isolated from Tilapia fish gill, harvested in sewage stabilization ponds in Tanzania and 5 O1 strains were isolated from two marine fish in Cochin, India during 2009–2011 (Kumar and Lalitha, 2013; Hounmanou et al., 2016). The presence of pathogenic serogroups O1 and O139 in fish scale samples collected in Mozambique have been detected by direct fluorescent antibody technique but the researchers were unable to culture them (Du Preez et al., 2010). In contrast, non-O1/O139 serogroups are prevalent worldwide in both freshwater and marine fish (Senderovich et al., 2010; Jones et al., 2013; Traor et al., 2014).

PCR based genotypic analysis revealed variability among the isolates, with 35 genotypic profiles comprising of 19 virulence factors (Table 2). Virulence factors other than cholera toxin are present in the isolates for example, both O1 and non-O1 strains were found to contain cholix toxin gene (*chxA*), a potent cytotoxin that is capable of halting protein synthesis in eukaryotic cells (Purdy et al., 2010) and the *stn/sto* gene for a heat-stable enterotoxin produced by toxigenic *V. cholerae* and *E. coli* (Rivera et al., 2001). Genes for the type III secretion system (TTSS) were detected in 18% of non-O1/O139 fish isolates. The major role of the TTSS in pathogenesis of non-O1/O139 *V. cholerae* induced diarrhea is already established (Dziejman et al., 2005). Infant rabbits orally inoculated with the wild type non-O1 strain AM-19226, which carries the gene for the TTSS, rapidly elicited a fatal diarrheal disease, and induced disruptions of the intestinal epithelium (Shin et al., 2011). Hemolysin, another virulence factor present in *V. cholerae*, promotes chloride secretion from intact human intestinal mucosa and capable of blood cell lysis in humans (Debellis et al., 2009). Reports indicate strains of non-O1/O139 *V. cholerae* isolates from hospitalized diarrheal patients in Kolkata, India, contained only the hemolysin (*hlyA*) gene, while negative for all other major toxin genes of *V. cholerae* (Chatterjee et al., 2009; Senderovich et al., 2010). The hemolysin gene (*hlyA*) was present in all fish isolates of this study. Another ubiquitously found virulence factor in this study was the type 6 secretion system (T6SS). Unterweger et al. (2012) reported that *V. cholerae* employs T6SS to compete commensal bacteria both in the human intestine and environment. The self-transmissible mobile genetic element termed the SXT element, have a crucial role in transferring antimicrobial drug resistance genes among microbial populations by conjugation (Toma et al., 2005). The SXT element of *V. cholerae* confers resistance to sulfamethoxazole, trimethoprim, chloramphenicol, and streptomycin (Waldor et al., 1996). In this study, 37 fish isolates contain the gene for the SXT element. Among them, 35 isolates showed resistance to at least one of the antibiotics tested, except for Chloramphenicol.

In the absence of CT and major colonization factors, culture supernatants of 4 non-toxigenic O1 (4 of 6 investigated) and 2 non-O1/O139 (2 of 3 investgated) strains showed a positve cytotoxic effect on HeLa cells by a mechanism which remains to be further characterized. Studies with non-O1/O139 strains showed a range of determinants for cytotoxicity, including hemolysins (Coelho et al., 2000), cholix toxin (Jørgensen et al., 2008), and heat stable enterotoxin (Arita et al., 1986). In this study, 4 cytotoxic strains possess *chxA*, 1 possesses *stn/sto* gene, and all the strains (n = 6) contained the *hlyA* gene. Eight of the nine strains showed a phenotype (fluid accumulation) in *in vivo* animal models similar to human disease despite the absence of major cholera toxin. Our results showed similar concordance with the previous reports of evoking fluid accumulation in the ileal loop test, despite lacking the CTX virulence cassette in *V. cholerae* O1 (Koley et al., 1999; Rajpara et al., 2013). Two non-O1/O139 fish strains possess a TTSS which mediates human diarrheal disease. Despite the high degree of virulence diversity, some fish strains showed genetic relatedness with pathogenic clones of diverse geographical locations. For example, the nontoxigenic O1 isolate F-91b fell in the same clade with toxigenic O1 N16961 and BX 330286 isolated from Bangladesh and Australia, respectively. These clonal relationships among fish and pandemic strains indicate that Hilsha fish may act as an environmental habitat where new pathogenic strains may emerge their non-pathogenic progenitors.

In conclusion, as cases of cholera in Bangladesh continue to occur, new transmission dynamics and their potential influence on virulence should be monitored. This study presents new data on the prevalence of *Vibrio cholerae* in Hilsha fish, and the possibility of an alternative route of transmission to households (as opposed to drinking water) in Bangladesh. The spectrum of the *V. cholerae* population isolated from Hilsha fish samples was highly heterogeneous, based on genotypic profile analyses. Nevertheless, the *Vibrio cholerae* isolates lacked cholera toxin, yet *in vitro* and *in vivo* activity showed the disease potential of the isolates. Despite the presence of the cholera toxin gene in Hilsha fish samples, isolation of toxigenic strains was not successful. Still it demands close monitoring of the coastal catch of Hilsha fish for cholera transmission and public health awareness to minimize the health risk posed by non-cholera *Vibrio* serogroups.

## Author contributions

ZH designed and carried out the study in the laboratory, analyzed the results and wrote the original draft. IF collected the samples, carried out the laboratory work and participated in acquisition of data. ST participated in critical reviewing and editing of original draft. PJ and AB conceived of the study and contributed to the revision of the draft and final approval of the version to be published. PJ was the principal supervisor of the project. All authors read and approved the final manuscript.

### Conflict of interest statement

The authors declare that the research was conducted in the absence of any commercial or financial relationships that could be construed as a potential conflict of interest.
