# Supplementary material for: Transmission and Toxigenic Potential of Vibrio cholerae in Hilsha Fish (Tenualosa ilisha) for Human Consumption in Bangladesh
Source: Front Microbiol. 2018 Feb 20;9:222. doi: 10.3389/fmicb.2018.00222 (PMC5826273; doi:10.3389/fmicb.2018.00222)
Supplement: Supplementary file 1 [file Table1.docx]

Supplementary Material

Transmission and toxigenic potential of *Vibrio cholerae* in Hilsha fish (*Tenualosa ilisha*) for human consumption in Bangladesh

**Zenat Zebin Hossain^1,2^, Israt Farhana^1^, Suhella Mohan Tulsiani^2,3^, Anowara Begum^1*^ and Peter Kjær Mackie Jensen^2,3^**

^1^ Department of Microbiology, University of Dhaka, Dhaka 1000, Bangladesh

^2^Institute of Public Health, University of Copenhagen, Copenhagen 1014, Denmark

^3^Copenhagen Centre for Disaster Research, University of Copenhagen, Copenhagen 1014, Denmark

*** Correspondence:**

Prof. Anowara Begum
[anowara@du.ac.bd](mailto:anowara@du.ac.bd)

**Supplementary Table 1:** Sampling locations of Hilsha fish

| Fish market name | Storage condition | GPS Coordinates |
| --- | --- | --- |
| Paturiaghat, Doulodia, Manikgonj | Freshly caught (without ice) | 23°47'08.3"N 89°48'43.4"E |
| Pagla bazaar, Narayanganj | Ice | 23°39'42.8"N 90°27'22.3"E |
| Ananadabazar, Palashi | Ice | 23°43'38.1"N 90°23'22.3"E |
| Boubazar 1,Arichpur | Ice | 23°53'11.1"N 90°24'22.3"E |
| Boubazar 2,Arichpur | Ice | 23°53'10.6"N 90°24'22.2"E |
| Kawran bazar | Ice | 23°45'09.5"N 90°23'40.1"E |
