# Supplementary material for: Transmission and Toxigenic Potential of Vibrio cholerae in Hilsha Fish (Tenualosa ilisha) for Human Consumption in Bangladesh
Source: Front Microbiol. 2018 Feb 20;9:222. doi: 10.3389/fmicb.2018.00222 (PMC5826273; doi:10.3389/fmicb.2018.00222)
Supplement: Supplementary file 2 [file Table2.docx]

Supplementary Material

Transmission and toxigenic potential of *Vibrio cholerae* in Hilsha fish (*Tenualosa ilisha*) for human consumption in Bangladesh

**Zenat Zebin Hossain^1,2^, Israt Farhana^1^, Suhella Mohan Tulsiani^2,3^, Anowara Begum^1*^ and Peter Kjær Mackie Jensen^2,3^**

^1^ Department of Microbiology, University of Dhaka, Dhaka 1000, Bangladesh

^2^Institute of Public Health, University of Copenhagen, Copenhagen 1014, Denmark

^3^Copenhagen Centre for Disaster Research, University of Copenhagen, Copenhagen 1014, Denmark

*** Correspondence:**

Prof. Anowara Begum
[anowara@du.ac.bd](mailto:anowara@du.ac.bd)

**Supplementary Table 2: Primers, probe and target genes in PCR experiment.**

| Target gene | Primer name | Sequence | Function | Amplicon size (bp) | Reference |
| --- | --- | --- | --- | --- | --- |
| *rfb O1* | O1 F | TCTATGTGCTGCGATTGGTG | ORF specific for *V. cholerae* O1 | 638 | ([Goel et al., 2007](#_ENREF_36)) |
|  | O1 R | CCCCGAAAACCTAATGTGAG |  |  |  |
| *rfb O139* | O139 F | AGCCTCTTTATTACGGGTGG | ORF specific for *V. cholerae* O139 | 449 | ([Hoshino et al., 1998](#_ENREF_45)) |
|  | O139 R | GTCAAACCCGATCGTAAAGG |  |  |  |
| *ctxB* | ctxB F | GGTTGCTTCTCATCATCGAACCAC | Intestinal receptor binding cholera toxin subunit B | 460 | ([Olsvik et al., 1993](#_ENREF_70)) |
|  | ctxB R | GATACACATAATAGAATTAAGGAT |  |  |  |
| *cep* | cep F | GCTACATGTTTAGCTCACTG | Core encoded pilus, a putative intestinal colonization factor of toxigenic *V. cholerae* | 251 | ([Bhattacharya et al., 2006](#_ENREF_12)) |
|  | cep R | TTTAGCCTTACGAATTAAGCC |  |  |  |
| *ace* | ace F | TAAGGATGTGCTTATGATGGACACCC | Accessory cholera enterotoxin, causes intestinal fluid secretion | 309 | ([Kumar et al., 2009](#_ENREF_59)) |
|  | ace R | CGTGATGAATAAAGATACTCATAGG |  |  |  |
| *tcp* | tcp F | CGTTGGCGGTCAGTCTTG | Toxin co-regulated pilus, essential intestinal colonization factor | 805 | ([Goel et al., 2007](#_ENREF_36)) |
|  | tcp R | CGGGCTTTCTTCTTGTTCG |  |  |  |
| *zot* | zot F | TCGCTTAACGATGGCGCGTTTT | Zonula Occludens Toxin, modifies intracellular tight junction | 947 | ([Singh et al., 2001](#_ENREF_89)) |
|  | zot R | AACCCCGTTTCACTTCTACCCA |  |  |  |
| *toxR* | toxR F | CCTTCGATCCCCTAAGCAATAC | Encodes a transcriptional activator controlling cholera toxin | 779 | ([Rivera et al., 2001](#_ENREF_80)) |
|  | toxR R | AGGGTTAGCAACGATGCGTAAG |  |  |  |
| *rtxC* | rtxC F | CGACGAAGATCATTGACGAC | Induces cytotoxic activity | 265 | ([Chow et al., 2001](#_ENREF_20)) |
|  | rtxC R | CATCGTCGTTATGTGGTTGC |  |  |  |
| *ompU* | ompU F | CCAAAGCGGTGACAAAGC | Outer membrane protein, a potential adherence factor and confers bile resistance | 655 | ([Karunasagar et al., 2003](#_ENREF_56); [Kumar et al., 2009](#_ENREF_59)) |
|  | ompU R | TTCCATGCGGTAAGAAGC |  |  |  |
| *hlyA* | hlyA F | GAGCCGGCATTCATCTGAAT | Encodes hemolysin toxin | 480 | ([Kumar et al., 2009](#_ENREF_59)) |
|  | hlyA R | CTCAGCGGGCTAATACGGTTTA |  |  |  |
| *mshA* | mshA F | AAAAGTCGACAGCGAAAGCGAATAGTGG | Encodes mannose- sensitive hemagglutinin pilus, a factor contributing to the ability of *V. cholerae* to adhere to plankton | 380 | ([Thelin and Taylor, 1996](#_ENREF_91); [Chiavelli et al., 2001](#_ENREF_18)) |
|  | mshA R | AAAAGGATCCATTGCACCAGCAACTGCACC |  |  |  |
| *chxA* | chxA F | TGGTGAAGATTCTCCTGCAA | Encodes a potent cytotoxin called Cholix toxin | 421 | ([Jørgensen et al., 2008](#_ENREF_53)) |
|  | chxA R | CTTGGAGAAATGGATGCGCTG |  |  |  |
| *HA-protease* | F | ACGTTAGTGCCCATGAGGTC | Encodes extracellur protease, which disrupts intestinal tight junctions by degrading protective mucus barrier | 350 | ([Haley et al., 2012](#_ENREF_39)) |
|  | R | ACGGCAAACACTTCAAAACC |  |  |  |
| *stn/sto* | F | TCG CAT TTA GCC AAA CAG TAG AAA | Encodes potent enterotoxin | 172 | ([Rivera et al., 2001](#_ENREF_80)) |
|  | R | GCT GGA TTG CAA CAT ATT TCG C |  |  |  |
| *SXT* | 1 | ATGGCGTTATCAGTTAGCTGGC | Encodes resistance  to sulfamethoxazole, trimethoprim, and streptomycin | 1035 | ([Bhanumathi et al., 2003](#_ENREF_11)) |
|  | 4 | GCGAAGATCATGCATAGACC |  |  |  |
| *vcsN2*(T3SS) | F | CAACACCTTCAAAGCCTTG | Encodes putative effector proteins which induce severe diarrhogenic mechanism in non-O1/O139 *V. cholerae* | 848 | ([Shin et al., 2011](#_ENREF_88); [Awasthi et al., 2013](#_ENREF_7)) |
|  | R | GCGAGCTCCAATTGAAAC |  |  |  |
| *vcsC2*(T3SS) | F | GGTCTCATAGACACTACG |  | 589 |  |
|  | R | ACGATGCTATGGGGTATG |  |  |  |
| *vopF*(T3SS) | F | GGAAATTCGCCAAGGTGTA |  | 839 |  |
|  | R | CAAAACCGTCCATACAAGG |  |  |  |
| *vasA*(T6SS) | F | GTACGACCGATCCTGACGTT | Accessory virulence factor which mediates protein translocation across cell membrane | 342 | ([Hasan et al., 2013](#_ENREF_42)) |
|  | R | ATCTGAATGGTCGTGGCTTC |  |  |  |
| *vasH* (T6SS) | F | GTGGCACGCTATTTCTGGAT |  | 385 |  |
|  | R | TTTCAGCTCACGCACATTTC |  |  |  |
| *vasK*(T6SS) | F | GCGTCAAATTCAGGAAGAGC |  | 399 |  |
|  | R | CTGTCCCAGAACCCAACTGT |  |  |  |
| *ctxA* | F | TTTGTTAGGCACGATGATGGAT | Major toxin to cause cholera | 84 | ([Blackstone et al., 2007](#_ENREF_14)) |
|  | R | ACCAGACAATATAGTTTGACCCACTAAG |  |  |  |
|  | Probe | **FAM**-TGTTTCCACCTCAATTAGTTTGAGAAGTGCCC-**BHQ1** |  |  |  |
