# Supplementary material for: Transmission and Toxigenic Potential of Vibrio cholerae in Hilsha Fish (Tenualosa ilisha) for Human Consumption in Bangladesh
Source: Front Microbiol. 2018 Feb 20;9:222. doi: 10.3389/fmicb.2018.00222 (PMC5826273; doi:10.3389/fmicb.2018.00222)
Supplement: Supplementary file 3 [file Table3.docx]

Supplementary Material

Transmission and toxigenic potential of *Vibrio cholerae* in Hilsha fish (*Tenualosa ilisha*) for human consumption in Bangladesh

**Zenat Zebin Hossain^1,2^, Israt Farhana^1^, Suhella Mohan Tulsiani^2,3^, Anowara Begum^1*^ and Peter Kjær Mackie Jensen^2,3^**

^1^ Department of Microbiology, University of Dhaka, Dhaka 1000, Bangladesh

^2^Institute of Public Health, University of Copenhagen, Copenhagen 1014, Denmark

^3^Copenhagen Centre for Disaster Research, University of Copenhagen, Copenhagen 1014, Denmark

*** Correspondence:**

Prof. Anowara Begum
[anowara@du.ac.bd](mailto:anowara@du.ac.bd)

**Supplementary Table 3: Control strains used in PCR experiment.**

| Organism name | Collection or isolation number | Source |
| --- | --- | --- |
| *V. cholerae* O1 | N16961 | Clinical |
| *V. cholerae* O1 | C6706 | Clinical |
| *V. cholerae* O139 | NIHC0270 | Clinical |
| *V. cholerae* non-O1 | 4460 | Clinical |
| *E. coli*^a^ | AN33859 | Clinical |

*Reference strains were collected from Laboratory of Molecular Genetics, International Centre for Diarrheal Disease Research, Bangladesh (ICDDR, B).

^a^ Used as negative control.
