# Supplementary material for: Transmission and Toxigenic Potential of Vibrio cholerae in Hilsha Fish (Tenualosa ilisha) for Human Consumption in Bangladesh
Source: Front Microbiol. 2018 Feb 20;9:222. doi: 10.3389/fmicb.2018.00222 (PMC5826273; doi:10.3389/fmicb.2018.00222)
Supplement: Supplementary file 4 [file Table4.docx]

Supplementary Material

Transmission and toxigenic potential of *Vibrio cholerae* in Hilsha fish (*Tenualosa ilisha*) for human consumption in Bangladesh

**Zenat Zebin Hossain^1,2^, Israt Farhana^1^, Suhella Mohan Tulsiani^2,3^, Anowara Begum^1*^ and Peter Kjær Mackie Jensen^2,3^**

^1^ Department of Microbiology, University of Dhaka, Dhaka 1000, Bangladesh

^2^Institute of Public Health, University of Copenhagen, Copenhagen 1014, Denmark

^3^Copenhagen Centre for Disaster Research, University of Copenhagen, Copenhagen 1014, Denmark

*** Correspondence:**

Prof. Anowara Begum
[anowara@du.ac.bd](mailto:anowara@du.ac.bd)

**Supplementary Table 4:** Accession numbers of *rpoB* sequences of 35 genotypic group representative *V. cholerae* isolates.

| Isolate ID | Virulence genotypic group | Accession Number |
| --- | --- | --- |
| F-32b | I | KX650640 |
| F-68b | II | KX710131 |
| F-32a | III | KX710135 |
| F-74b | IV | KX710123 |
| F-51a | V | KX710124 |
| F-48a | VI | KX710121 |
| F-36a | VII | KX650642 |
| F-44 | VIII | KX710118 |
| F-53 | IX | KX710126 |
| F-72 | X | KX710132 |
| F-56d | XI | KX710136 |
| F-6 | XII | KU568172 |
| F-69 | XIII | KX710137 |
| F-1b | XIV | KU568169 |
| F-1a | XV | KT779427 |
| F-2 | XVI | KU568170 |
| F-16b | XVII | KX650635 |
| F-5 | XVIII | KX894541 |
| F-49d | XIX | KX710122 |
| F-73 | XX | KX710138 |
| F-85 | XXI | KX710133 |
| F-9 | XXII | KU568175 |
| F-47 | XXIII | KX710120 |
| F-63c | XXIV | KX710130 |
| F-42a | XXV | KX650644 |
| F-52a | XXVI | KX710125 |
| F-56a | XXVII | KX710128 |
| F-55a | XXVIII | KX710127 |
| F-17 | XXIX | KX650636 |
| F-19 | XXX | KX650637 |
| F-34c | XXXI | KX650641 |
| F-21 | XXXII | KX650638 |
| F-23b | XXXIII | KX650639 |
| F-22 | XXXIV | KX710134 |
| F-61 | XXXV | KX710129 |
